# Association of community sanitation usage with soil-transmitted helminth infections among school-aged children in Amhara Region, Ethiopia

**DOI:** 10.1186/s13071-017-2020-0

**Published:** 2017-02-17

**Authors:** William E. Oswald, Aisha E. P. Stewart, Michael R. Kramer, Tekola Endeshaw, Mulat Zerihun, Berhanu Melak, Eshetu Sata, Demelash Gessese, Tesfaye Teferi, Zerihun Tadesse, Birhan Guadie, Jonathan D. King, Paul M. Emerson, Elizabeth K. Callahan, Matthew C. Freeman, W. Dana Flanders, Thomas F. Clasen, Christine L. Moe

**Affiliations:** 1 Department of Disease Control, London School of Hygiene and Tropical Medicine, London, UK; 2 Department of Epidemiology, Emory University, Atlanta, GA USA; 3 The Carter Center, Atlanta, GA USA; 4 The Carter Center, Addis Ababa, Ethiopia; 5 Amhara Regional Health Bureau, Bahir Dar, Ethiopia; 6 World Health Organization, Geneva, Switzerland; 7 International Trachoma Initiative, Atlanta, GA USA; 8 Department of Environmental Health, Emory University, Atlanta, GA USA; 9 Hubert Department of Global Health and Center for Global Safe Water, Sanitation, and Hygiene, Emory University, Atlanta, GA USA

**Keywords:** Sanitation, Soil-transmitted helminths, Ethiopia

## Abstract

**Background:**

Globally, in 2010, approximately 1.5 billion people were infected with at least one species of soil-transmitted helminth (STH), *Ascaris lumbricoides*, *Trichuris trichiura*, hookworm (*Ancylostoma duodenale* and *Necator americanus*). Infection occurs through ingestion or contact (hookworm) with eggs or larvae in the environment from fecal contamination. To control these infections, the World Health Organization recommends periodic mass treatment of at-risk populations with deworming drugs. Prevention of these infections typically relies on improved excreta containment and disposal. Most evidence of the relationship between sanitation and STH has focused on household-level access or usage, rather than community-level sanitation usage. We examined the association between the proportion of households in a community with latrines in use and prevalence of STH infections among school-aged children.

**Methods:**

Data on STH prevalence and household latrine usage were obtained during four population-based, cross-sectional surveys conducted between 2011 and 2014 in Amhara, Ethiopia. Multilevel regression was used to estimate the association between the proportion of households in the community with latrines in use and presence of STH infection, indicated by > 0 eggs in stool samples from children 6–15 years old.

**Results:**

Prevalence of STH infection was estimated as 22% (95% CI: 20–24%), 14% (95% CI: 13–16%), and 4% (95% CI: 4–5%) for hookworm, *A. lumbricoides*, and *T. trichiura*, respectively. Adjusting for individual, household, and community characteristics, hookworm prevalence was not associated with community sanitation usage. *Trichuris trichuria* prevalence was higher in communities with sanitation usage ≥ 60% *versus* sanitation usage < 20%. Association of community sanitation usage with *A. lumbricoides* prevalence depended on household sanitation. Community sanitation usage was not associated with *A. lumbricoides* prevalence among households with latrines in use. Among households without latrines in use, *A. lumbricoides* prevalence was higher comparing communities with sanitation usage ≥ 60% *versus* < 20%. Households with a latrine in use had lower prevalence of *A. lumbricoides* compared to households without latrines in use only in communities where sanitation usage was ≥ 80%.

**Conclusions:**

We found no evidence of a protective association between community sanitation usage and STH infection. The relationship between STH infection and community sanitation usage may be complex and requires further study.

**Electronic supplementary material:**

The online version of this article (doi:10.1186/s13071-017-2020-0) contains supplementary material, which is available to authorized users.

## Background

Globally, in 2010, approximately 1.5 billion people were infected with at least one species of soil-transmitted helminth (STH) [1]. The four most common nematode worms that infect humans are: the roundworm, *Ascaris lumbricoides*; the whipworm, *Trichuris trichiura*; and, the hookworms, *Ancylostoma duodenale* and *Necator americanus* [2, 3]. These intestinal parasites infect humans through exposure to eggs or larvae that develop in the environment after being deposited in feces [4]. Eggs and larvae thrive in warm, moist soils of the tropics and subtropics, particularly in poorer areas with inadequate access to sanitation [3, 4]. Recent estimates suggest that among 800 million people in sub-Saharan Africa, 130 million are infected with hookworm, 50 million with *A. lumbricoides*, and 37 million people with *T. trichiura* [5].

STH infections infrequently lead to mortality, but chronic infection results in several detrimental outcomes, including impaired physical and cognitive development, school absenteeism and poor performance, reduced work productivity among adults, adverse pregnancy outcomes, anemia, and possibly increased susceptibility to malaria, tuberculosis, and HIV [2, 3]. The extent of morbidity is related to the burden of infection, the number of worms residing within the host, and the health of the host [3, 6].

Current strategies for control of STH in low-income countries focus on large-scale provision of anthelmintic drugs to prevent the consequences of chronic infection [2, 7]. The World Health Organization (WHO) recommends periodic administration of albendazole and mebendazole to at-risk populations, including preschool-aged children, school-aged children, women of reproductive age, including pregnant women and lactating mothers, and other groups with high exposure [7]. It is recognized that long-term control based on deworming efforts through mass treatment need to be complemented with concurrent improvements in sanitation and excreta disposal behaviors [3, 8–12].

Using data from population-based surveys and associated stool samples collected between 2011 and 2014, we estimated the association between the proportion of households in a community with latrines in use and prevalence of STH infections among children, aged 6 to 15 years, in Amhara, Ethiopia. We hypothesized that higher community sanitation usage would be associated with lower prevalence of these STH infections.

## Methods

### Study participants and overview

**Fig. 1:**
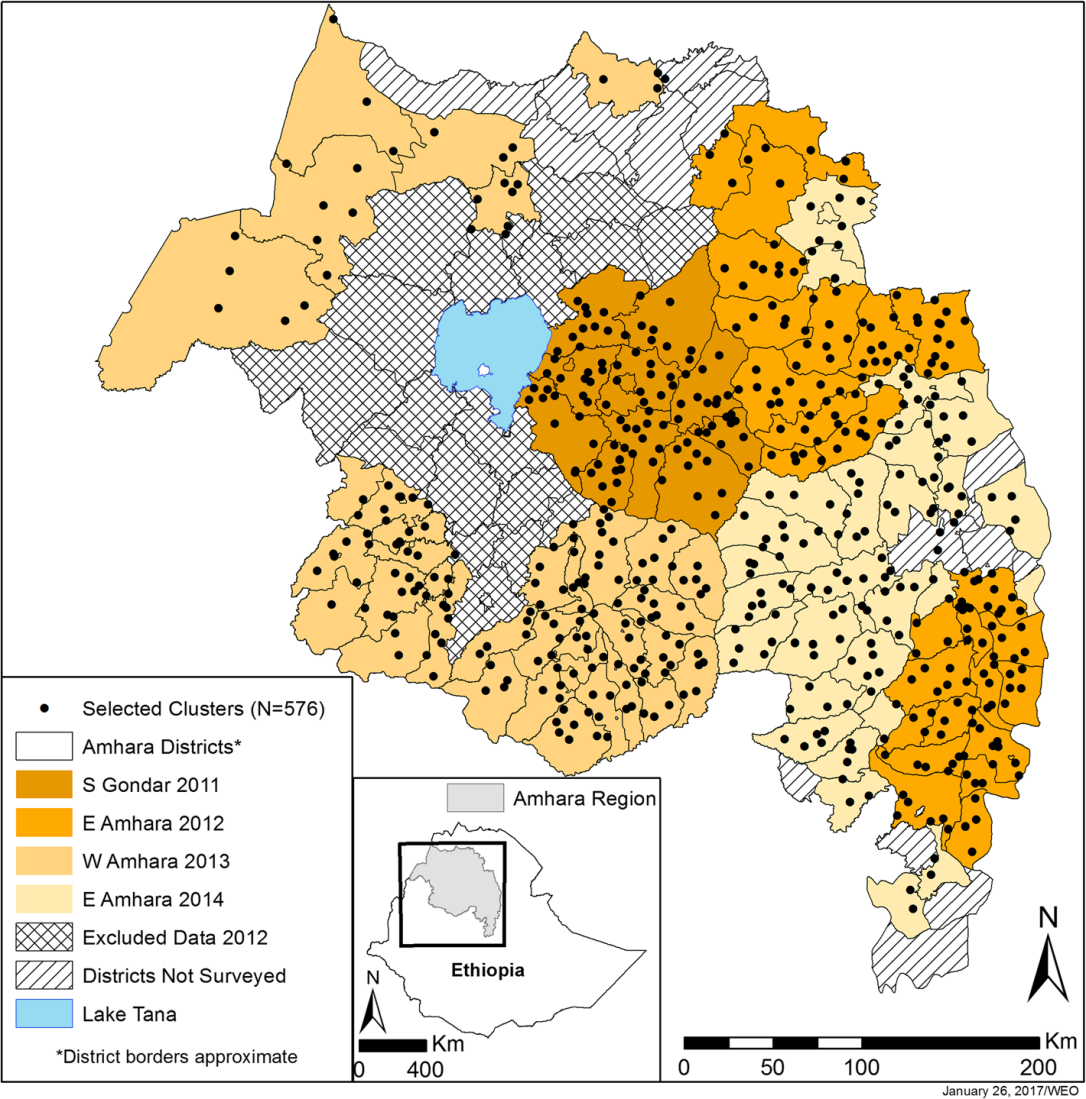
Location of clusters and districts, by survey and year, Amhara Region, Ethiopia, 2011–2014

For this analysis, data were combined from four population-based, cross-sectional surveys conducted in distinct areas of Amhara between 2011 and 2014. Data from an additional survey conducted in North Gondar and West Gojjam zones between May and June 2012 were excluded because of data quality concerns (Fig. 1). The methods and results of these surveys have been described previously [13–15]. Briefly, surveys used a multi-stage cluster random sampling methodology and were powered to estimate zonal prevalences of STH infections, including *A. lumbricoides* (AL), *T. trichiura* (TT), and hookworm (HW). ‘Woreda’ (Ethiopian administrative units equivalent to districts) became eligible for surveying when at least five rounds of annual azithromycin mass drug administration for trachoma control had occurred. The smallest administrative units with population data available are ‘gott’ (villages) and were primary sampling units. Within each eligible district, villages were listed by geographical distribution and systematically selected probability proportional to population size (Median village size: 205 households; IQR: 23–1055). Within villages, smaller administrative units of approximately 40 households, called development teams (DT), were used as segments for a modified segment survey design [16, 17]. Development teams were listed upon arrival in the community with an appropriate village representative, who then drew numbers from a hat to select DTs to be surveyed. In villages of 40 households or less, the entire village was surveyed. For the current study, selected DTs were considered clusters, the immediate geographic area of residence of participants.

In selected clusters, village leaders were interviewed for community information. Heads of all households were interviewed for demographic and socioeconomic information and knowledge and practices regarding trachoma, water, sanitation, and hygiene. Visual inspections were made of household latrines and handwashing stations. Within each cluster, one child aged 6 to 15 years old (2 to 15 years in 2011 survey) was randomly selected in each household, after enumerating all residents, and asked to provide a single stool sample. Interviews were conducted with selected children about school attendance, use of latrines for defecation, receipt of anthelmintic treatment, recent infection with worms, and shoe wearing. Responses were recorded electronically using tablet computers operating Swift Insights software (The Carter Center, Atlanta, GA, USA) [18].

### Exposure and outcome measures

The exposure, community sanitation usage, was calculated as the proportion of households within the cluster with a latrine to which there was a defined path and feces were observed in the pit [19].

Stool sampling methods, training, and quality control have been described previously [13]. An ether-concentration method was used to enumerate the number of helminth eggs per 1 gram of stool, fixed in 10 ml of sodium acetate-acetic acid-formalin (SAF) solution, counting from 1 to 99 eggs and then recording ≥ 100 if higher [20, 21]. Outcomes were dichotomous indicators for presence of > 0 eggs of each species (AL, HW, TT) in stool samples. Frequencies of infection intensity were tabulated across categories of community sanitation usage.

### Covariates

Individual measures included child’s age in years (centered at 10), sex, and reported usually attending school. Between 2011 and subsequent surveys, some questions were asked differently, so to avoid missing values the following approaches were used to combine responses. In 2011, reported wearing of shoes was recorded as: (i) always; (ii) sometimes; or (iii) never. Subsequent surveys recorded whether the child was observed to be currently wearing shoes. For an indicator of shoe wearing, responses from 2011 of always wearing shoes were combined with positive observations of shoe wearing in subsequent surveys. In 2011, the child and parent/guardian were asked whether the child had received and taken albendazole or mebendazole: (i) in the past month; (ii) between 1 month and 1 year; or (iii) 1 year ago. The first two responses for either medication were combined to indicate receipt of medication within the last year. For a measure of recent anthelmintic treatment, responses to the 2011 question were combined with responses to the question from subsequent surveys of whether the child had taken medicine for worms in the last year. In 2011, reported use of a latrine by the child was recorded as: (i) always; (ii) sometimes; or (iii) never. In subsequent surveys, children were asked if they last defecated in a school latrine, family latrine, open field, or backyard. For an indicator of latrine usage, responses from 2011 of always using a latrine were combined with responses in subsequent surveys of last defecating in a school or the family’s latrine. Children were also asked if they had worms in the last year.

Household access to water was dichotomized < 30 min or not, based on asking how long it took to fetch water for bathing. Reported type of drinking water source was dichotomized as improved or not according to WHO/UNICEF Joint Monitoring Programme classification [22]. Indicators were created for presence of a pit latrine and presence of a pit latrine in use (defined above). Household wealth was indicated by ownership of radio, television, mobile phone, metal roof, and access to electricity. A categorical variable was created for the highest level of education completed by respondents in 2011 or by any household member in subsequent surveys.

Cluster wealth was calculated as the mean total of reported wealth indicators per household. Mean elevation in meters was calculated for each cluster from household measurements and evaluated as a continuous measure. Population density (km^-2^) in 2011 was generated using the Oak Ridge National Laboratory’s LandScan as an unprojected map in WGS84 with 83.33 × 10^−4^° resolution [23]. Annual average volumetric soil moisture (m^3^/m^3^) measures for 2010, produced by the European Space Agency Climate Change Initiative (ESA CCI), were obtained as a grid file in WGS84 with a Lambert Azimuthal Equal Area projection and 0.25° resolution [24]. Population density and soil moisture values were extracted for each cluster using geographic coordinates in ArcMap 10.1 (ESRI, Redlands, CA, USA). Population density was evaluated as a continuous measure, natural log transformed, and dichotomized at 250 people km^-2^. Soil moisture was evaluated as a continuous measure. Soil moisture measures for 18 clusters were unavailable because of their proximity to Lake Tana. Each cluster was assigned the nearest neighbor’s value. Presence of a health post, health center, or hospital was dichotomized as presence of any health facility.

### Analyses

Means and frequencies were estimated with confidence intervals across categories of community sanitation usage, accounting for study design and sampling weights, based on inverse total selection probability for clusters (village and DT) and individuals. *F*-statistics were calculated using an adjusted Wald test for categorical variables and Analysis of Variance for continuous measures. Multilevel Poisson regression with robust variance was used to estimate the association between proportion of households in each cluster with a latrine in use and infection with each of three species of soil-transmitted helminths among children aged 6 to 15 years. Modified Poisson regression uses robust error variance to correct overestimation of error when applied to binomial data and allows direct estimation of prevalence ratios (PR) [25, 26]. Potential confounders, among measures recorded in all surveys, were identified based on literature review. Reported measures for child’s school attendance, location of last defecation, and having worms in past year were not modeled. An evaluation of directed acyclic graphs (DAGs) identified the same minimal sufficient set of covariates to estimate associations of community sanitation usage with each STH infection [27, 28]. A sequential modeling approach, removing covariates at each level from fully-adjusted models, was also used to identify confounders based on changes in exposure estimates. All models controlled for survey round to account for year and possible differences. Results are presented from crude, DAG-based, and fully-adjusted models for comparison.

Generalized linear mixed models were fit, specifying a random intercept for cluster and incorporating sampling weights. Robust standard errors were requested to account for clustering within districts, and adaptive quadrature with eight integration points was used. Results are reported for individual weights scaled to sum to the cluster sample size, though weights were also scaled to effective cluster sample size for comparison [29]. Operationalization of exposure as a categorical measure, *versus* linear or quadratic, was based on a preliminary assessment considering fit and interpretability. Participants missing covariates in any survey were excluded from models. Effect modification on the multiplicative scale of the association of community sanitation usage with STH infection by household latrine use, anthelmintic treatment, and by wearing of shoes (for HW infection) was evaluated with Wald tests. Measures of association were presented for community sanitation usage within strata of each potential effect modifier, as stratified prevalence ratios with a single reference category, and for household sanitation within strata of community sanitation [30]. An analysis to further examine robustness of estimates of the association of household sanitation with AL infection within strata of community sanitation usage is described in Additional file 1: Supplementary information. Individual and cluster mean shoe wearing were assessed as negative control exposures *a posteriori* to detect uncontrolled confounding of the association of community sanitation usage with AL and TT infection [31]. All described analyses were conducted using Stata 13.1 (StataCorp LP, College Station, TX, USA).

## Results

### Characteristics of the study population

Of 14,417 children selected, stool sample results were obtained for 12,754 children (88%). The combined dataset linked community, household, and individual information and complete parasitological results for 11,009 (76%) children aged 6 to 15 years in 576 clusters in 133 districts (Fig. 1). The analysis included 9818 (89%) observations with complete results for AL and TT and 9812 (89%) observations for HW.

Table 1 describes individual, household, and community characteristics of children aged 6 to 15 years, overall and by community sanitation usage category. Children in communities with lower sanitation usage had indicators of less household education and access to health facilities, worse access to water for bathing and drinking, and more impoverished and less densely-populated living conditions, compared to children in communities with higher sanitation usage. Among school-aged children, 65% (95% CI: 61–69%) and 85% (95% CI: 82–87%) reported attending school in communities with lowest and highest sanitation usage, respectively. Of children’s households, 38% (95% CI: 30–46%) and 63% (95% CI: 54–72%) reported an improved source of drinking water, comparing communities with lowest and highest sanitation usage respectively. Households in communities with lowest sanitation usage had a mean of 0.68 items (95% CI: 0.59–0.76) compared to 1.67 items (95% CI 1.47–1.87) in communities with highest sanitation usage.

**Table 1:** Individual, household, and community characteristics of 11,009 children aged 6 to 15 years by community proportion of households with latrines in use in Amhara Region, Ethiopia, 2011–2014

% Households with latrines in use		0– < 20%		20– < 40%		40– < 60%		60– < 80%		80–100%		Total		
	*n*	Mean (%)	95% CI	Mean (%)	95% CI	Mean (%)	95% CI	Mean (%)	95% CI	Mean (%)	95% CI	Mean (%)	95% CI	*P* ^a^
Children, aged 6–15 years (*n*)		2472		1837		2202		2467		2031		11,009		
Communities (*n*)		137		97		109		125		108		576		
Hookworm	11,009	0.24	0.20–0.29	0.18	0.13–0.24	0.20	0.16–0.26	0.23	0.18–0.28	0.24	0.19–0.30	0.22	0.20–0.24	0.47
*T. trichiura*	11,009	0.03	0.02–0.04	0.03	0.01–0.06	0.04	0.03–0.06	0.06	0.04–0.09	0.07	0.05–0.10	0.04	0.04–0.05	0.01
*A. lumbricoides*	11,009	0.10	0.07–0.13	0.16	0.12–0.21	0.14	0.11–0.18	0.16	0.12–0.20	0.17	0.13–0.20	0.14	0.13–0.16	0.04
Age, years	10,961	9.68	9.53–9.82	10.02	9.86–10.17	10.08	9.93–10.23	10.12	9.98–10.26	10.28	10.10–10.46	10.02	9.96–10.09	<0.01
Male sex	11,000	0.48	0.46–0.51	0.49	0.46–0.52	0.50	0.47–0.52	0.48	0.46–0.51	0.45	0.42–0.47	0.48	0.47–0.49	0.09
Reported usually attending school	10,833	0.65	0.61–0.69	0.72	0.66–0.76	0.80	0.76–0.83	0.79	0.76–0.82	0.85	0.82–0.87	0.76	0.74–0.78	<0.01
Reported always wearing shoes (2011)	1029	0.06	0.01–0.19	0.02	0.00–0.08	0.10	0.02–0.39	0.04	0.01–0.14	0.30	0.14–0.53	0.09	0.05–0.17	<0.01
Any observed shoes (No 2011)	9795	0.46	0.40–0.52	0.57	0.49–0.66	0.51	0.43–0.59	0.43	0.36–0.50	0.48	0.40–0.55	0.48	0.46–0.51	0.13
Any reported or observed shoes (All)	10,824	0.42	0.37–0.47	0.52	0.44–0.60	0.49	0.42–0.57	0.41	0.35–0.48	0.47	0.40–0.54	0.46	0.44–0.48	0.18
Anthelmintics in past year (2011)	1016	0.09	0.05–0.18	0.06	0.02–0.15	0.16	0.05–0.43	0.12	0.06–0.23	0.27	0.14–0.44	0.13	0.09–0.18	0.06
Anthelmintics in past year (No 2011)	9224	0.17	0.12–0.22	0.17	0.12–0.23	0.14	0.11–0.18	0.21	0.16–0.26	0.20	0.15–0.26	0.18	0.16–0.20	0.28
Anthelmintics in past year (All)	10,240	0.16	0.12–0.21	0.16	0.11–0.21	0.14	0.11–0.18	0.20	0.16–0.26	0.21	0.16–0.27	0.17	0.15–0.20	0.19
Reported always use latrine (2011)	1026	0.05	0.02–0.12	0.18	0.11–0.28	0.41	0.23–0.61	0.58	0.42–0.73	0.92	0.81–0.97	0.36	0.27–0.46	<0.01
Reported last use of latrine (No 2011)	9643	0.10	0.08–0.13	0.37	0.32–0.42	0.56	0.52–0.60	0.77	0.73–0.80	0.88	0.84–0.91	0.54	0.51–0.57	<0.01
Reported use of latrine (All)	10,669	0.10	0.08–0.12	0.35	0.30–0.40	0.55	0.51–0.59	0.76	0.72–0.79	0.88	0.85–0.91	0.53	0.50–0.55	<0.01
Worms in past year	10,251	0.34	0.29–0.39	0.35	0.29–0.42	0.33	0.27–0.39	0.36	0.32–0.41	0.37	0.31–0.43	0.35	0.32–0.37	0.85
Household owns latrine	10,986	0.13	0.11–0.16	0.43	0.39–0.46	0.63	0.60–0.65	0.81	0.79–0.82	0.95	0.94–0.96	0.59	0.56–0.61	<0.01
Household owns latrine in use	10,963	0.09	0.07–0.11	0.37	0.35–0.39	0.57	0.55–0.59	0.77	0.75–0.78	0.93	0.92–0.95	0.54	0.52–0.57	<0.01
Bathing water access < 30 min	10,769	0.49	0.41–0.56	0.57	0.48–0.65	0.62	0.53–0.70	0.71	0.64–0.78	0.68	0.59–0.75	0.61	0.58–0.65	<0.01
Improved drinking water source	10,792	0.38	0.30–0.46	0.53	0.42–0.63	0.56	0.46–0.64	0.55	0.46–0.63	0.63	0.54–0.72	0.52	0.48–0.56	<0.01
Household has														
Radio	10,770	0.12	0.09–0.14	0.14	0.10–0.20	0.21	0.17–0.25	0.23	0.19–0.26	0.25	0.21–0.31	0.19	0.17–0.21	<0.01
TV	10,772	0.00	0.00–0.02	0.02	0.01–0.04	0.02	0.01–0.04	0.05	0.03–0.08	0.09	0.06–0.14	0.04	0.03–0.05	<0.01
Electricity	10,766	0.01	0.00–0.03	0.07	0.03–0.15	0.08	0.04–0.14	0.19	0.13–0.27	0.22	0.15–0.30	0.11	0.09–0.14	<0.01
Mobile phone	10,766	0.16	0.13–0.21	0.24	0.19–0.30	0.30	0.26–0.35	0.28	0.23–0.34	0.32	0.27–0.38	0.26	0.24–0.28	<0.01
Iron roof	10,783	0.45	0.40–0.51	0.57	0.49–0.65	0.68	0.62–0.74	0.76	0.70–0.80	0.75	0.69–0.81	0.64	0.62–0.67	<0.01
Highest education of an adult	10,774													<0.01
None		0.53	0.47–0.58	0.49	0.42–0.56	0.42	0.37–0.49	0.39	0.34–0.43	0.36	0.30–0.43	0.44	0.41–0.46	
Religious		0.03	0.02–0.05	0.02	0.01–0.03	0.03	0.02–0.05	0.05	0.03–0.07	0.03	0.02–0.05	0.03	0.03–0.04	
Primary school		0.20	0.17–0.25	0.14	0.11–0.18	0.19	0.15–0.23	0.19	0.15–0.23	0.22	0.17–0.27	0.19	0.17–0.21	
Junior secondary		0.15	0.12–0.17	0.21	0.17–0.25	0.21	0.18–0.24	0.19	0.17–0.22	0.21	0.18–0.26	0.19	0.18–0.20	
Senior secondary		0.03	0.02–0.04	0.07	0.05–0.09	0.10	0.08–0.12	0.12	0.10–0.14	0.12	0.09–0.15	0.09	0.08–0.10	
College/University		0.00	0.00–0.01	0.01	0.01–0.03	0.01	0.01–0.02	0.02	0.02–0.04	0.03	0.02–0.04	0.02	0.01–0.02	
Non-formal education		0.05	0.04–0.08	0.06	0.04–0.09	0.04	0.03–0.05	0.05	0.03–0.07	0.03	0.02–0.05	0.05	0.04–0.05	
Mean total of wealth indicators per household	576	0.68	0.59–0.76	0.92	0.78–1.07	1.23	1.08–1.37	1.45	1.28–1.61	1.67	1.47–1.87	1.19	1.12–1.26	<0.01
Population density (km^−2^)	574	451	206–695	414	198–629	405	237–573	1013	544–1481	1459	896–2023	764	596–933	<0.01
Has a health facility	553	0.08	0.04–0.14	0.19	0.12–0.29	0.23	0.16–0.33	0.18	0.12–0.26	0.30	0.21–0.40	0.19	0.16–0.23	<0.01
Elevation (m)	575	2043	1962–2124	2259	2140–2379	2263	2178–2348	2359	2250–2468	2317	2220–2415	2244	2215–2273	<0.01
Soil moisture (m^3^/m^3^)	575	0.25	0.24–0.26	0.26	0.25–0.27	0.26	0.25–0.28	0.29	0.28–0.29	0.29	0.28–0.30	0.27	0.27–0.27	<0.01

*Abbreviation*: *CI* confidence interval

^a^ *P*-values from Wald adjusted F-test for categorical variables or ANOVA *F*-test for difference in continuous means

Children’s shoe wearing and treatment with anthelmintics were not associated with community sanitation usage (*F*_(3.86,1789.08)_ = 1.56, *P* = 0.1833 and *F*_(3.92,1813.85)_ = 1.54, *P* = 0.1879, respectively). Compared to communities with higher sanitation usage, communities with lower sanitation usage were in areas with lower population density (*F*_(4,460)_ = 4.49, *P* = 0.0014) and lower elevation (*F*_(4,460)_ = 6.77, *P* < 0.0001). Soil moisture was significantly lower in communities with lower sanitation usage compared to communities with higher sanitation usage (*F*_(4,460)_ = 11.47, *P* < 0.0001), but the magnitude of difference may not reflect meaningful change.

In 576 clusters, mean community sanitation usage was 50% (95% CI: 47–52%) and ranged from 0% in 44 clusters (8%) to 100% in 14 clusters (2%). HW was the most prevalent of these STH across surveyed areas of Amhara, infecting almost a quarter of school-aged children (Table 1: 22%, 95% CI: 20–24%). TT was least prevalent, infecting 4% of school-aged children (Table 1: 95% CI: 4–5%).

Table 2 presents results from crude and adjusted models of the association of community sanitation usage with prevalence of each STH, controlling for selected covariates and survey. Results were generally robust to sampling weight scaling method (data not shown), but potentially meaningful identified differences between weighted and unweighted results are discussed (Additional file 1: Tables S1 and S2).

**Table 2:** Association of infection with hookworm, *Trichuris trichiura* and *Ascaris lumbricoides* with community proportion of households with latrines in use and household ownership of latrine in use among children aged 6 to 15 years in Amhara Region, Ethiopia, 2011–2014

Infection	Sanitation measure		Crude^a^		DAG-based^b^		Full^c^	
			aPR	95% CI	aPR	95% CI	aPR	95% CI
Hookworm	Community	≥ 80%	0.80	0.54–1.18	1.21	0.82–1.80	1.19	0.81–1.77
	% Households with latrines in use per cluster	60– < 80%	0.67	0.46–0.98	1.05	0.70–1.56	1.03	0.70–1.52
		40– < 60%	0.77	0.51–1.16	1.01	0.69–1.50	0.99	0.68–1.44
		20– < 40%	0.69	0.46–1.03	0.88	0.59–1.32	0.87	0.59–1.29
		< 20%	Ref		Ref		Ref	
	Household							
	Latrine in use		–	–	–	–	1.00	0.91–1.08
	No latrine in use		–	–	–	–	Ref	
*T. trichiura*	Community	≥ 80%	4.12	1.80–9.42	3.56	1.39–9.14	3.70	1.40–9.76
	% Households with latrines in use per cluster	60– < 80%	2.82	1.23–6.48	2.50	1.01–6.21	2.50	1.02–6.14
		40– < 60%	2.08	0.96–4.53	2.01	0.90–4.49	2.10	0.95–4.63
		20– < 40%	0.91	0.39–2.12	0.89	0.38–2.08	0.89	0.39–2.05
		< 20%	Ref		Ref		Ref	
	Household							
	Latrine in use		–	–	–	–	0.93	0.78–1.11
	No latrine in use		–	–	–	–	Ref	
*A. lumbricoides*	Community	≥ 80%	2.48	1.58–3.90	2.33	1.42–3.84	2.35	1.37–4.01
	% Households with latrines in use per cluster	60– < 80%	2.02	1.28–3.19	1.81	1.12–2.92	1.80	1.09–2.98
		40– < 60%	1.47	0.98–2.21	1.45	0.98–2.16	1.44	0.95–2.16
		20– < 40%	1.62	1.02–2.58	1.49	1.01–2.20	1.47	0.99–2.18
		< 20%	Ref		Ref		Ref	
	Household							
	Latrine in use		–	–	–	–	1.01	0.89–1.14
	No latrine in use		–	–	–	–	Ref	

Models for *A. lumbricoides* and *T. trichuris* included data on 9818 children in 574 communities. Models for hookworm included data on 9812 children in 574 communities

*Abbreviations*: *aPR* adjusted prevalence ratio, *CI* confidence interval. Results weighted to account for unequal probabilities of selection

^a^Crude model only controlled for survey round

^b^DAG-based model was adjusted for elevation; population density; community mean total of wealth indicators per household; soil moisture; and survey round

^c^Full model for *A. lumbricoides* and *T. trichuris* adjusted for age; sex; anthelmintic treatment; bathing water source < 30 min; improved drinking water source; household owns: radio, television, mobile phone, iron roof, and has access to electricity; household education; elevation; soil moisture; community mean total of wealth indicators per household; population density; and survey round. Full model for hookworm adjusted for the same covariates in addition to shoe wearing.

### Hookworm infection

Community sanitation usage ≥ 20% was associated with lower HW prevalence, compared to usage of < 20%, adjusting only for survey. Based on the crude model, the difference was statistically significant, only where usage was between 60– < 80% (PR 0.67, 95% CI: 0.46–0.98). Adjusting for potential confounders in both DAG-based and full models attenuated the association towards or past the null across usage categories. In the full model, adjusting for community sanitation usage and other factors, household ownership of a latrine in use was not associated with hookworm prevalence (PR 1.00, 95% CI: 0.91–1.08).

### *Trichuris trichiura* infection

TT prevalence in communities with sanitation usage ≥ 40% was more than double the prevalence in communities with sanitation usage of < 20%. In the crude model, community sanitation usage was significantly associated with elevated prevalence of TT at usage ≥ 60%, compared to usage < 20%. Estimates from DAG-based and full model were not meaningfully different. After adjusting for all potential confounders in the full model, community sanitation usage ≥ 60% was significantly associated with higher prevalence of TT, compared to usage < 20 (60– < 80%, PR 2.50, 95% CI: 1.02–6.14; ≥ 80%, PR 3.70, 95% CI: 1.40–9.76). Adjusting for community sanitation usage and other factors, household ownership of a latrine in use was not significantly associated with TT prevalence (PR 0.93, 95% CI: 0.78–1.11). When included in full models as negative control exposures, individual shoe wearing was associated with TT infection, but the association was not statistically significant (PR 0.79, 95% CI: 0.61–1.03). Cluster mean shoe wearing was not associated with TT infection (PR 1.04, 95% CI: 0.93–1.16).

### *Ascaris lumbricoides* infection

Based on the crude model, community sanitation usage of ≥ 20% was associated with higher prevalences of AL compared to usage of < 20%. Estimates from DAG-based and full model were not meaningfully different. Adjusting for all potential confounders moderately attenuated estimated associations, and community sanitation usage ≥ 60% was significantly associated with higher prevalence of AL, compared to usage < 20 (Full: 60– < 80%, PR 1.80, 95% CI: 1.09–2.98; ≥80%, PR 2.35, 95% CI: 1.37–4.01). Adjusting for community sanitation usage and other factors, household ownership of a latrine in use was not associated with AL prevalence (PR 1.01, 95% CI: 0.89–1.14). Variables for shoe wearing, individually and aggregated to cluster, were not associated with AL infection when included in full models as negative control exposures (Individual: PR 0.96, 95% CI: 0.84–1.09; Cluster mean reported/observed, PR 0.96, 95% CI: 0.91–1.02).

### Effect modification

Reported receipt of deworming treatment in the past year did not significantly modify the association of community sanitation usage with any of the infections (data not shown). Measures of shoe wearing did not significantly modify the association between community sanitation usage and hookworm infection (data not shown).

Table 3 shows prevalence ratios comparing children in respective strata of community and household sanitation usage. No significant modification by household latrine usage of the association of community sanitation usage with HW (*P* = 0.15) or TT (*P* = 0.40) prevalence was detected. The association between community sanitation usage and AL prevalence was significantly modified by household latrine usage, adjusting for all covariates (*P* < 0.01). The first two groups of columns compare prevalences of AL between communities with sanitation usage ≥ 20% to those with usage < 20%, among children from households with and without latrines in use. Community sanitation usage was not associated with AL prevalence among children from households with latrines in use. Children from households without latrines in use had increasingly higher prevalences of AL when comparing communities with higher sanitation usage to communities with usage < 20% (PR Range: 1.41–3.92). Examining the joint association of increased community sanitation and a household latrine in use, children in households with a latrine in use in communities with any level of sanitation usage had higher prevalences of AL compared to children in households without latrines in use in communities with sanitation usage < 20% (PR Range: 1.40–2.36).

**Table 3:** Association of hookworm, *Trichuris trichiura* and *Ascaris lumbricoides* with community sanitation usage by household latrine use among children aged 6 to 15 years in Amhara Region, Ethiopia, 2011–2014

	% Households with latrines in use per cluster	Community sanitation by household sanitation						Joint association			
		Latrine in use			No latrine in use			Latrine in use		Household sanitation by community sanitation	
Infection		+/−	PR	95% CI	+/−	PR	95% CI	PR	95% CI	PR^a^	95% CI
Hookworm	≥ 80%	335/1315	1.15	0.68–1.96	23/93	1.22	0.80–1.87	1.19	0.81–1.75	0.98	0.77–1.24
	60– < 80%	300/1316	1.03	0.61–1.73	90/448	0.91	0.60–1.38	1.06	0.72–1.56	1.16	0.97–1.40
	40– < 60%	181/956	0.91	0.54–1.52	180/698	1.05	0.73–1.52	0.94	0.64–1.38	0.89	0.80–1.00
	20– < 40%	81/514	0.82	0.48–1.38	149/891	0.89	0.60–1.32	0.84	0.56–1.28	0.95	0.78–1.16
*P*_interaction_ = 0.15	< 20%	33/154	Ref		445/1610	Ref		1.03	0.76–1.41	1.03	0.76–1.41
*T. trichiura*	≥ 80%	123/1527	3.30	0.99–11.02	9/107	2.60	0.81–8.30	3.59	1.42–9.08	1.38	0.88–2.17
	60– < 80%	93/1525	2.08	0.68–6.32	34/504	2.90	1.20–7.03	2.26	0.90–5.65	0.78	0.56–1.08
	40– < 60%	52/1085	1.85	0.66–5.22	42/836	2.09	0.91–4.79	2.02	0.90–4.49	0.97	0.70–1.33
	20– < 40%	13/583	0.78	0.25–2.38	28/1012	0.90	0.39–2.05	0.85	0.33–2.19	0.94	0.60–1.46
*P*_interaction_ = 0.40	< 20%	7/180	Ref		48/2010	Ref		1.09	0.49–2.40	1.09	0.49–2.40
*A. lumbricoides*	≥ 80%	256/1394	1.68	0.93–3.06	30/86	3.92	2.11–7.27	2.36	1.45–3.85	0.60	0.44–0.81
	60– < 80%	255/1363	1.31	0.74–2.34	89/449	2.03	1.24–3.33	1.84	1.14–2.98	0.91	0.76–1.08
	40– < 60%	173/964	1.12	0.67–1.87	124/754	1.41	0.90–2.19	1.57	1.05–2.34	1.11	0.85–1.47
	20– < 40%	99/497	1.13	0.65–1.96	166/874	1.50	1.00–2.25	1.58	1.06–2.35	1.06	0.80–1.39
p_interaction_ < 0.01	< 20%	28/159	Ref		188/1870	Ref		1.40	1.00–1.96	1.40	1.00–1.96

Results weighted to account for unequal probabilities of selection. Models for *A. lumbricoides* and *T. trichuris* included data on 9818 children in 574 communities and adjusted for age; sex; anthelmintic treatment; bathing water source < 30 min; improved drinking water source; household owns: radio, television, mobile phone, iron roof, and has access to electricity; household education; elevation; soil moisture; community mean total of wealth indicators per household; population density; and survey round. Model for hookworm included data on 9812 children in 574 communities and adjusted for the same covariates in addition to shoe wearing.

*Abbreviations*: +/−, Number with/without infection, *PR* prevalence ratio, *CI* confidence interval

^a^PR, Prevalence ratio for household latrine in use *versus* household latrine not in use, within strata of community sanitation usage; p_interaction_, Global Wald

The last column in Table 3 compares prevalences between children from households with and without latrines in use by community sanitation usage. In communities with sanitation usage ≥ 80%, children in households with a latrine in use had significantly lower prevalence of AL compared to children in households without a latrine in use (≥ 80%, PR 0.60, 95% CI: 0.44–0.81); while in communities with sanitation usage < 20%, children in households with a latrine in use had higher prevalence of AL compared to children in households without a latrine in use (< 20%, PR 1.40, 95% CI: 1.00–1.96).

### Infection intensity

Intensities of infection with each worm as measured by eggs per gram in stool samples were very low in this population and showed a similar pattern as prevalence (Table 4). Only intensity of infection with TT was significantly associated with community sanitation usage (*F*_(6.43,2979.09)_ = 2.97, *P* = 0.0055).

**Table 4:** Intensity (eggs per gram) of infection by community proportion of households with latrines in use among children aged 6 to 15 years in Amhara Region, Ethiopia, 2011–2014

		Community sanitation usage												
		< 20%		20– < 40%		40– < 60%		60– < 80%		≥ 80%		Total		
Infection	Eggs/gram	*n*	%	*n*	%	*n*	%	*n*	%	*n*	%	*n*	%	*P* ^a^
Hookworm	0	1919	0.76	1536	0.83	1794	0.79	1975	0.77	1597	0.76	8821	0.78	0.47
	1–49	490	0.22	254	0.17	359	0.20	410	0.21	377	0.23	1890	0.21	
	50–99	24	0.01	3	0.00	10	0.01	18	0.01	18	0.01	73	0.01	
	≥ 100	9	0.00	3	0.00	5	0.00	8	0.00	5	0.00	30	0.00	
*T. trichiura*	0	2382	0.97	1753	0.97	2069	0.96	2279	0.94	1858	0.93	10,341	0.96	< 0.01
	1–49	55	0.02	41	0.03	93	0.04	129	0.06	133	0.07	451	0.04	
	50–99	1	0.00	1	0.00	3	0.00	2	0.00	3	0.00	10	0.00	
	≥ 100	4	0.00	1	0.00	3	0.00	1	0.00	3	0.00	12	0.00	
*A. lumbricoides*	0	2205	0.91	1510	0.84	1836	0.86	2024	0.84	1660	0.83	9235	0.86	0.13
	1–49	175	0.07	193	0.11	215	0.09	272	0.11	220	0.11	1075	0.10	
	50–99	33	0.01	43	0.02	59	0.02	57	0.02	57	0.03	249	0.02	
	≥ 100	29	0.01	50	0.02	58	0.02	58	0.02	60	0.03	255	0.02	

^a^ *P*-values from Wald adjusted *F*-test for categorical variables

## Discussion

Our findings show no evidence that increased community sanitation usage was protective against the three most-common STH infections among children aged 6 to 15 years in Amhara Region, Ethiopia. These findings contrast with current understanding of the relationship between sanitation and STH infection, and the relationship between community sanitation and these STH remains unclear. Much of the evidence of the relationship between sanitation and STH infection has focused on household sanitation access or usage, rather than community sanitation. Two recent meta-analyses examined accumulated evidence of the relationship between sanitation and STH infection and found protective associations of household sanitation access with lower odds of any STH, AL, TT, and HW infection [32, 33]. In their systematic review, Ziegelbauer et al. [33] identified only six studies that examined community sanitation and STH infection. Two recent studies from Tanzania found that higher community sanitation coverage was associated with lower prevalence odds of AL and weakly associated with higher prevalence odds of HW, controlling for individual, household, and environmental measures [34, 35].

We observed no association of community or household sanitation with HW prevalence, after controlling for individual, household, and community characteristics. Infection intensity (represented by egg counts) directly represents transmission rate because no STH reproduction occurs within the host [2]. As an indicator of transmission, frequencies of HW infection intensities did not significantly differ across categories of community sanitation usage (Table 4). Hookworm may live up to seven years in the gut [2]. In the absence of deworming, which was infrequent in this population, it is perhaps not unusual that a reduction in prevalence was not observed for HW within the latrines’ times in place, which was less than 3 years on average (data not shown) [36].

There was little relative difference in AL prevalence by community sanitation usage among children in households with latrines in use. It is understood that most AL transmission clusters within households and families [37, 38]. A study from Bangladesh found that household-related exposures explained 58% of clustering of AL worm burden at the household level, indicating the importance of the domestic domain in transmission [38]. Therefore, not finding a significant association between community sanitation usage and AL prevalence in this population subset is less surprising [6]. Among children from households without latrines in use, AL prevalence increased with greater community sanitation usage relative to communities with lowest sanitation usage. This subset of children resided in households that were last to adopt household sanitation in their communities, which might indicate an increased likelihood of worse hygiene conditions or practices related to other AL transmission routes.

A significant protective association of sanitation with AL prevalence was observed among children from households with latrines in use compared to children from households without latrines in use among communities with sanitation usage ≥ 80%. This result corresponds with odds ratios observed in recent meta-analyses of 0.62 (95% CI: 0.44–0.88) and 0.78 (95% CI: 0.60–1.00), representing reductions in the odds of AL infection with household sanitation use [32, 33]. Our finding could indicate that household latrines may only be protective against AL at specific levels of community sanitation usage. A study in Tanzania found a non-significant protective association of household latrine ownership when community latrine coverage was included in the model, but each 10% increase in latrine coverage was associated with a reduction in AL prevalence odds [35]. Community sanitation usage is not frequently reported in studies of household sanitation, so further studies are warranted to confirm this finding.

Among communities with low sanitation usage, children in households with a latrine in use had significantly higher AL prevalence compared to children in households without a latrine in use. The magnitude of the association was smaller with exclusion of sampling weights (Additional file 1: Table S1), so this result should be interpreted with caution. A plausible explanation for the finding may be that in communities with fewer latrines overall, there is increased reliance on sharing sanitation infrastructure between families. A recent systematic review found a consistent pattern of elevated risk of helminth infection among those relying on shared sanitation facilities [39]. Curtale et al. [40] and Tshikuka et al. [41] found that increased numbers of users and sharing increased intensity of AL infections. Shared sanitation is not currently included in the definition of improved sanitation because facilities may not be accessible at all times and poor cleanliness may not fully separate users from contact with human waste [39]. Information on latrine cleanliness and maintenance was not collected, so further exploration of the mechanism behind this possible transmission was not possible. Future studies should collect information on latrine sharing, particularly in contexts with limited sanitation availability, and indicators of latrine construction, maintenance, and cleanliness to explore these possible transmission pathways.

Our dataset allowed for characterization of each child’s immediate and community environment. As an evaluation activity, however, limited information could be collected during household surveys. Our outcome measure was based on a single, small sample of stool, so prevalence may have been underestimated because egg excretion varies by day and egg distribution is not uniform in stool [42]. Our indicator of household latrine usage balanced standard recommendations with the logistical realities of program evaluation, but the aggregated measure for community sanitation usage may not sufficiently reflect levels of fecal contamination in the environment. For example, there was no actual measure of consistent latrine usage by all household members or measures of child feces disposal and hand hygiene. The difficulty with accurately measuring sanitation usage has been acknowledged [43]. Furthermore, as a cross-sectional study, the possibility that latrine promotion activities were targeted to areas with higher STH prevalences cannot be ruled out.

Models controlled for potential confounders among available measures and other possible differences between survey rounds. Additional unmeasured factors were controlled through application of remote-sensing information, but residual confounding is possible with any observational study. TT prevalence and infection intensity were observed to increase with increasing community sanitation usage (Tables 2 and 4), but household ownership of a latrine in use was not associated with lower prevalence of TT, adjusting for other factors. Overall prevalences of AL were higher in communities with highest sanitation usage. Community sanitation usage may reflect unmeasured factors related to urbanization that were not completely controlled by included measures. Urban areas are generally believed to have higher prevalences of AL and TT compared to rural areas [44]. In their review, Brooker et al. [4] found no consistent pattern of differences between urban and rural communities for the prevalence of AL and TT among a limited number of studies, but concluded that hookworm appeared equally prevalent in rural and urban settings.

Our statistical models adjusted for population density using a remote-sensing derived measure. This measure of population density, along with our other included measures, may not have adequately controlled for confounding related to urbanization. To identify residual confounding, individual and cluster mean shoe wearing were included in DAG-based and fully-adjusted models for AL and TT infection as negative control exposures [31]. If these control exposures do not cause AL and TT infection and have a comparable set of confounders as community sanitation usage, then any detected association of these exposures with the outcomes would indicate bias in the main association of interest [31]. Under the necessary assumptions of comparability between these measures of shoe wearing and community sanitation, our results did not strongly indicate the presence of any residual confounding with AL. There was some indication of residual confounding of the association of community sanitation usage and TT based on the indicator for individual shoe wearing.

## Conclusions

In the current study, we found no evidence of a protective association between community sanitation usage and STH infection and evidence of a protective association with household sanitation only for AL under conditions of high community sanitation usage. Sanitation may convey other private and public benefits, including convenience, dignity, privacy, and safety [45]. The extent of sanitation usage in this study reflects promising uptake of sanitation in the Amhara Region, but reductions in STH prevalence may still require additional improvements in sanitation-related behaviors to substantially reduce exposure to fecal contamination.

## Additional file

Additional file 1: Supplementary information. **Table S1.** Estimated measures of association of household ownership of a latrine in use with prevalence of *Ascaris lumbricoides* infection, using conditional logistic regression and mixed regression with and without individual sampling weights. **Table S2.** Number of clusters (*n*) contributing to each stratum-specific analysis out of the total number of clusters within that stratum of community sanitation usage (*N*) and correlation between cluster-specific odds ratios of association of household ownership of a latrine in use with prevalence of *Ascaris lumbricoides* infection and respective sampling weight by strata of community sanitation usage. (DOCX 24 kb)
