# Supplementary material for: Association of community sanitation usage with soil-transmitted helminth infections among school-aged children in Amhara Region, Ethiopia
Source: Parasit Vectors. 2017 Feb 17;10:91. doi: 10.1186/s13071-017-2020-0 (PMC5316142; doi:10.1186/s13071-017-2020-0)
Supplement: Additional file 1: — Supplementary information. Table S1. Estimated measures of association of household ownership of a latrine in use with prevalence of Ascaris lumbricoides infection, using conditional logistic regression and mixed regression with and without individual sampling weights. Table S2. Number of clusters (n) contributing to each stratum-specific analysis out of the total number of clusters within that stratum of community sanitation usage (N) and correlation between cluster-specific odds ratios of association of household ownership of a latrine in use with prevalence of Ascaris lumbricoides infection and respective sampling weight by strata of community sanitation usage. (DOCX 24 kb) [file 13071_2017_2020_MOESM1_ESM.docx]

**Additional file 1**

**Supplementary information**

*Introduction*

Modification of the effect of community sanitation usage on *Ascaris lumbricoides* (AL) infection prevalence by household sanitation usage was examined by testing the significance of product terms in the regression model. Product terms included an indicator for household ownership of a latrine in use and indicators for four categories of community sanitation usage. A Wald test was used to assess whether interaction was significant. Prevalence ratios were examined for the effect of community sanitation within strata of household sanitation, and for household sanitation within strata of community sanitation. Interestingly, the association of household sanitation was significantly protective (PR 0.60, 95%CI 0.44--0.81) only in the highest category of community sanitation but non-protective in the lowest category of community sanitation (PR 1.40, 95%CI 1.00--1.96). The finding of a non-protective effect in communities with low overall sanitation usage, though biologically plausible, warranted further examination.

*Conditional logistic regression*

A conditional logistic regression was conducted in SAS 9.4 (SAS Institute Inc., Cary, NC) to estimate the association of household sanitation with AL prevalence conditioning on cluster and survey, within each of the categories of community sanitation usage. The overall pattern of the results were comparable to those from the original random effects model with some differences.

**Table S1. Estimated measures of association of household ownership of a latrine in use with prevalence of *Ascaris lumbricoides* infection, using conditional logistic regression and mixed regression with and without individual sampling weights.**

| **% Households with latrines in use per cluster** | **Conditional Logistic Regression** | | | | **Mixed Model** | | | |
| --- | --- | --- | --- | --- | --- | --- | --- | --- |
|  | **Conditioning on cluster and survey** | | | |  |  |  |  |
|  | **Crude** | | **Adjusted*** | | **Adjusted**** | | **Adjusted**** | |
|  | **Unweighted** | | **Unweighted** | | **Unweighted** | | **Ind. Weights, Scaled A** | |
|  | **OR** | **95% CI** | **OR** | **95% CI** | **PR** | **95% CI** | **PR** | **95% CI** |
|  |  |  |  |  |  |  |  |  |
| **≥80%** | 0.44 | 0.28–0.70 | 0.42 | 0.26–0.70 | 0.57 | 0.44–0.75 | 0.60 | 0.44–0.81 |
| **60-<80%** | 0.74 | 0.56–0.98 | 0.90 | 0.66–1.23 | 0.92 | 0.78–1.09 | 0.91 | 0.76–1.08 |
| **40-<60%** | 1.05 | 0.80–1.38 | 1.01 | 0.75–1.35 | 1.03 | 0.81–1.30 | 1.11 | 0.85–1.47 |
| **20-<40%** | 1.03 | 0.75–1.41 | 1.03 | 0.73–1.44 | 1.03 | 0.81–1.30 | 1.06 | 0.80–1.39 |
| **<20%** | 1.06 | 0.63–1.76 | 1.15 | 0.66–1.98 | 1.20 | 0.87–1.65 | 1.40 | 1.00–1.96 |
|  |  |  |  |  |  |  |  |  |

OR=Odds Ratio; PR=Prevalence Ratio; CI=Confidence Interval

*Adjusted for age; sex; anthelmintic treatment; bathing water source < 30 min; improved drinking water source; household owns: radio, television, mobile phone, iron roof, and has access to electricity; household education

**Adjusted for age; sex; anthelmintic treatment; bathing water source < 30 min; improved drinking water source; household owns: radio, television, mobile phone, iron roof, and has access to electricity; household education; elevation; soil moisture; community mean total of wealth indicators per household; population density; and survey round

Table S1 shows the unadjusted odds ratios (ORs) and ORs adjusted for individual and household factors from the conditional logistic regression, conditioning on cluster and survey and not incorporating sampling weights. The protective association of household sanitation with AL prevalence in the highest community sanitation category was even more pronounced in this unweighted analysis (Crude: OR 0.44, 95%CI 0.28--0.70; Adjusted: OR 0.42, 95%CI 0.26--0.70) than observed in the mixed model. However, the non-protective association in the lowest community sanitation category was more attenuated (Crude: OR 1.06, 95%CI 0.63--1.76; Adjusted: OR 1.15, 95%CI 0.66--1.98).

The ORs from these conditional logistic regression models were similar to prevalence ratios obtained from the mixed model that did not include sampling weights (Table S1), adjusting for individual, household, and community level factors (Sanitation usage ≥80%: PR 0.57, 95%CI 0.44--0.75; Sanitation usage <20%: PR 1.20, 95%CI 0.87--1.65). The difference between results from unweighted and weighted mixed models highlights the potential influence of sampling weights on stratum-specific estimated measures of association.

*Correlation between cluster-specific odds ratios and sampling weights*

Correlation between the cluster-specific ORs, or measures of association, and sampling weights was examined to assess their possible influence on our results. Sampling weights should not be associated with the magnitude of association measures, but if strong measures of association have higher weights or *vice versa*, then it could explain the difference between unweighted and weighted results.

To examine this, ORs were calculated per cluster for the association between household ownership of a latrine in use (yes/no) and each child’s AL infection status (yes/no). ORs of 0 and infinity resulting from non-marginal 0 counts were included as 0 and 99, respectively. The correlation between cluster ORs and respective sampling weights was examined with Spearman correlation coefficients and scatterplots with a fitted regression line.

**Table S2. Number of clusters (n) contributing to each stratum-specific analysis out of the total number of clusters within that stratum of community sanitation usage (N) and correlation between cluster-specific odds ratios of association of household ownership of a latrine in use with prevalence of *Ascaris lumbricoides* infection and respective sampling weight by strata of community sanitation usage.**

| **% Households with latrines in use per cluster** |  |  |  |  | **% Households with latrines in use per cluster** | **Mean Individual Weights** | | **Cluster Weights** | |
| --- | --- | --- | --- | --- | --- | --- | --- | --- | --- |
|  | **n** | **N** | **%** |  |  | **Rho*** | **p** | **Rho*** | **p** |
|  |  |  |  |  |  |  |  |  |  |
| **≥80%** | 55 | 108 | 0.51 |  | **≥80%** | 0.10 | 0.45 | 0.11 | 0.42 |
| **60-<80%** | 92 | 125 | 0.74 |  | **60-<80%** | -0.05 | 0.64 | -0.02 | 0.85 |
| **40-<60%** | 74 | 109 | 0.68 |  | **40-<60%** | 0.03 | 0.80 | 0.06 | 0.63 |
| **20-<40%** | 64 | 97 | 0.66 |  | **20-<40%** | 0.00 | 0.99 | -0.07 | 0.59 |
| **<20%** | 47 | 137 | 0.34 |  | **<20%** | 0.26 | 0.08 | 0.07 | 0.66 |
|  |  |  |  |  |  |  |  |  |  |

*Spearman correlation coefficient for cluster OR association with respective sampling weights

Many of the clusters within strata of community sanitation usage dropped out when calculating the cluster-stratified OR because of marginal 0 counts. Table S2 shows the number of clusters (n) contributing to each stratum-specific analysis out of the total number of clusters within that category of community sanitation usage (N). The lowest and highest categories have the lowest proportions of contributing clusters. This finding makes sense as there are fewer households with and without latrines in use within the categories of lowest and highest community sanitation usage, respectively.

Table S2 shows the Spearman correlation coefficients between cluster-specific ORs for the association of household ownership of a latrine in use with AL prevalence and mean individual sampling weights by category of community sanitation usage. The individual sampling weights were marginally associated with the magnitude of cluster ORs in the lowest category of community sanitation usage (Rho=0.26, p=0.08). Cluster sampling weights were not associated with OR magnitudes.

In the lowest category of community sanitation usage, there is evidence of marginally significant correlation between cluster OR magnitude and mean individual sampling weight, but there was one cluster with a high mean individual weight (~320) and a high OR (~20) that could increase the magnitude of the estimated association within the lowest category of community sanitation usage when weights were incorporated.

*Conclusion*

Based on these findings, a non-protective association of household sanitation was observed within the lowest stratum of community sanitation usage, but the magnitude of this association was strongly influenced by the individual weights in each cluster and the limited number of clusters contributing to the analysis. The conclusion of a non-protective association is presented but with this limitation.
